# Translational Potential of an Electrospun Polycaprolactone Scaffold for Anterior Cruciate Ligament Reconstruction

**DOI:** 10.1007/s42765-025-00632-8

**Published:** 2025-11-03

**Authors:** Jinrong Lin, Kaili Chen, Meng Liang, Tania Choreno Machain, Daisy Crouch, Simona Mengoli, George Exley, Alma Zaplluzha, Mathew Baldwin, William Jackson, Thomas Cosker, Sarah Snelling, Andrew Carr, Gordon Blunn, Andrew Price, Pierre-Alexis Mouthuy

**Affiliations:** 1 Nuffield Department of Orthopaedics, Rheumatology and Musculoskeletal Sciences, Botnar Research Centre, University of Oxford, Oxford OX3 7LD, UK; 2 Nuffield Orthopaedic Centre, Oxford University Hospitals NHS Foundation Trust, Oxford OX3 7LD, UK; 3 School of Pharmacy and Biomedical Sciences, Faculty of Science and Health, University of Portsmouth, St Michael’s Building, White Swan Road, Portsmouth PO1 2DT, UK

**Keywords:** Anterior cruciate ligament (ACL), Poly(ε-caprolactone) (PCL), Artificial ligament, Electrospun scaffold, Braiding

## Abstract

Anterior cruciate ligament (ACL) injuries are common and often require surgical reconstruction. Autografts remain the clinical standard for ACL reconstruction (ACLR) but are limited by donor site morbidity, inconsistent outcomes, and supply constraints. Here, we report the development of electrospun ligament (ES-Lig), a fully degradable, electrospun scaffold composed of poly(ε-caprolactone) (PCL) designed to mimic the extracellular matrix (ECM) of the native ACL. A scalable manufacturing process was established, incorporating electrospinning, filament stretching, alignment, and braiding. ES-Lig demonstrated controlled in vitro degradation over 12 months while retaining sufficient mechanical strength for early-stage healing. Mechanical characterisation revealed tensile properties and fixation stability comparable to autografts. In vitro biocompatibility was confirmed through cytotoxicity assays, patient-derived ACL explants, and direct cell growth onto the material. In an ovine ACLR model, ES-Lig enabled functional recovery, tissue infiltration throughout its length, and joint stability within 10 weeks post-implantation. Histological and imaging analyses confirmed graft-bone integration, vascularisation, and early ligamentisation. These findings establish ES-Lig as a promising, clinically translatable scaffold for next-generation ACL repair.

## 1 Introduction

Ligaments are fibrous connective tissues essential for joint stability and movement [1]. Among them, the anterior cruciate ligament (ACL) is one of the most frequently injured, with an estimated annual incidence of 30 to 78 cases per 100,000 people, and rising rates particularly among adolescents [2, 3]. If left untreated, ACL injuries can lead to functional impairment, reduced quality of life, and an increased risk of osteoarthritis [4]. In the United Kingdom, ACL injuries impose an annual financial burden of approximately £63 million [5]. Traditionally, ACL reconstruction (ACLR) with autografts, such as hamstring tendon or bone patellar tendon bone grafts, has been considered the gold standard [6]. However, this approach is associated with high donor site morbidity [7], prolonged rehabilitation [8], and a failure rate of 13.4% overall [9], rising to 21% in athletes under 25 years [10].

Artificial grafts have emerged as a potential option to address the limitations of autografts in ACLR. Most efforts to date have focused on non-degradable synthetic polymers like nylon [11], polyethylene terephthalate [12], and polyester [13], which offer advantages in mechanical strength and manufacturing feasibility. Nevertheless, these materials present both short- and long-term challenges, including device rejection, chronic tissue inflammation, and permanent elongation [11, 14]. More importantly, their inability to support cellular infiltration and extracellular matrix (ECM) deposition compromises graft-to-bone healing and ligament regeneration [15], often resulting in bone tunnel enlargement and graft failure that need revision surgeries [16]. To overcome these limitations, there is an increasing emphasis on developing biomaterials that possess biocompatibility, biodegradability, and high initial mechanical strength to support neo-ligament formation while mitigating long-term immune response. Biological materials like silk have been explored [17], but their clinical translation has been limited due to inferior mechanical properties and immunogenicity concerns [18]. In contrast, degradable synthetic polymers, particularly those processed via electrospinning, have shown greater potential [19].

Electrospinning, a technique that creates fibrous structures resembling the ECM, has shown promise in supporting desirable cellular behaviours such as proliferation, migration, and differentiation [20, 21]. Native ligament is organised as a complex ECM, where nanometre-sized collagen fibrils align into micrometre-scale fibres and fascicles to bear load [22]. Inspired by the native ECM’s multi-scale collagen network, our group previously developed a method for the production of continuous filaments that are sufficiently strong to undergo various textile processes such as twisting, weaving and braiding [23–26]. While initially demonstrated with poly(p-dioxanone) (PDO), a fast-degrading polymer, this approach can be extended to other polymers such as poly(ε-caprolactone) (PCL), a biocompatible polymer used in many United States Food and Drug Administration (FDA)-approved medical devices [27]. With higher mechanical strength and a slower degradation rate of two to four years, PCL is a particularly suitable candidate for ACLR applications [20, 26]. Previous studies have shown that electrospun PCL bundles can achieve a Young’s modulus comparable to the native ACL and support proliferation, infiltration, and lineage-specific differentiation of mesenchymal stem cells [28–30]. However, these grafts were generally limited to single- or multi-bundle constructs produced at laboratory scale. Building on our earlier demonstration of weaving PCL electrospun yarns [31], our goal was to establish a braiding strategy to generate textile-based PCL grafts that better mimic the native ACL structure and can be produced in a scalable, automatable manner for translational use.

In this study, we established a robust and scalable manufacturing process for producing electrospun ligament (ES-Lig), a braided PCL-based artificial graft engineered to mimic the ECM of the native ACL. The scale-up was achieved through a series of optimised steps, including electrospinning, stretching, multifilament alignment, and final braiding, all of which can be implemented in an automated system. ES-Lig exhibited gradual in vitro degradation while retaining mechanical properties comparable to autografts and sufficient to support early tissue formation. Biocompatibility assays and patient-derived ACL explant models confirmed sustained cell viability and progressive cellular infiltration within the scaffold. Building on these results, in vivo implantation in an ovine ACLR model further validated ES-Lig’s performance, showing stable graft fixation, host tissue integration, and functional recovery by 10 weeks.

## 2 Experimental Section

### 2.1 Fabrication of ES-Lig

The entire ES-Lig manufacturing process was conducted under International Organization for Standardization (ISO) Class 7 cleanroom conditions at the Botnar Institute for Musculoskeletal Sciences (NDORMS, University of Oxford), with real-time environmental monitoring (temperature, humidity, pressure, and particulate levels) to ensure product cleanliness and consistency.

#### 2.1.1 Preparation of PCL Solution for Electrospinning

Medical-grade PCL resin (Ashland Specialities Ireland Ltd.) was dissolved in 1,1,1,3,3,3-hexafluoroisopropanol (HFIP; DKSH Marketing Services Spain, S.A.U.; Halocarbon Product Corporation) at a concentration of 10% (w/v). The solution was agitated at room temperature on a roller mixer at 15 r/min for 24 h. The fully dissolved PCL solution was then used for the subsequent electrospinning process.

#### 2.1.2 Electrospinning of Continuous PCL Filaments

Continuous PCL filaments were electrospun from the pre-mixed polymer solution onto a wire collector (100 µm in diameter; Goodfellow Cambridge Ltd, Huntingdon, UK), adjusting from a previously described protocol [20, 31]. Electrospinning was carried out using a single-nozzle setup comprising a high-voltage power supply system (World Precision Instruments Ltd., Florida, USA) and a syringe pump (Harvard Apparatus Ltd., Massachusetts, USA). Both the nozzle and the wire collector were positioned inside a glove box under constant airflow to remove HFIP vapours generated during the process. The wire collector was cleaned with 70% ethanol prior to use. The distance between the nozzle and the wire collector was about 17 cm, with an applied voltage of an average of 6 kV. The speed of the wire collector was maintained at 1.44 m/h.

#### 2.1.3 Stretching of the Electrospun Filaments

Electrospun filaments were stretched to achieve a final length 600–800% greater than their original length, corresponding to draw ratios ranging from 1:6 to 1:8. Stretching was performed using a drawing machine, and knots were applied to maintain filament continuity upon filament breakage. The drawn filaments were collected onto a new spool and stored in a desiccator for the subsequent alignment step.

#### 2.1.4 Alignment of Stretched Filaments

Nine stretched filaments were drawn together to form a single multifilament bundle using a twisting machine (Marui Textile Machine Co., Ltd., Osaka, Japan). The aligned filaments were collected onto a new spool at a rotation speed of 50 r/min.

#### 2.1.5 Braiding of the Cord as ES-Lig

Braided cord production was carried out using a braiding machine (VF 1/4–32-140, Herzog GmbH). The process employed 24 carriers with bobbins, prepared by evenly redistributing the previously aligned multifilament bundles. The machine was operated at a speed of 25 r/min with a lay length of 30 mm. A continuous braided cord was produced and subsequently cut into 300 mm segments, each corresponding to a single ES-Lig unit.

#### 2.1.6 Packaging, and Ethylene Oxide (EtO) Sterilisation of ES-Lig

The braided cord was placed into custom die-cut inserts (Riverside Medical Centre, Kankakee, USA), which were packaged and sealed in Tyvek® pouches. Packaged samples underwent EtO sterilisation (Anderson Caledonia Ltd, Bellshill, UK), with temperature and humidity monitored throughout. After sterilisation, units were placed in a drying chamber to decrease humidity before final sealing, completing the preparation of sterile, ready-to-use ES-Lig products.

### 2.2 Characterisation of Filaments and ES-Lig

Surface morphology of electrospun and stretched filaments was examined using scanning electron microscopy (SEM). Samples were mounted on stubs with carbon conductive adhesive tape and gold-coated using a sputter coater (SC7620 Mini, Quorum Technologies Ltd., UK). Imaging was performed on an environmental SEM (EVO LS15, Carl Zeiss, Germany) at different magnifications. Tensile testing was conducted using a Zwick tensile machine (Zwick Roell Group, Germany). Filament samples (10 cm in length) were tested at 0.5 mm/min, and ES-Lig cords (10 cm in length) at 160 mm/min, until failure (*n* = 3). Force at break (N) and elongation (mm) were recorded. Micro-computed tomography (MicroCT) imaging was employed to view the cross-section of ES-Lig. Gel permeation chromatography (GPC) was outsourced to evaluate the molecular weight of PCL at different time points.

### 2.3 In vitro Degradation Analysis and Neutral Red Uptake (NRU) Cytotoxicity Assay

In vitro degradation of ES-Lig and individual filaments was performed in phosphate-buffered saline (PBS, pH 7.4) at 37 °C for up to 12 months. All samples were either sterilised in 70% ethanol for 24 h over two cycles and subsequently dried or further subjected to EtO sterilisation. Samples were prepared at uniform lengths and with comparable initial weights. At predetermined time points (0, 1, 3, 6, and 12 months), mass loss, breaking force via tensile testing, and molecular weight were measured and recorded. The NRU assay was conducted following the guidelines outlined in British Standard/European Norm/International Organization for Standardization (BS EN ISO) 10993-5:2009. For the assay, ES-Lig and polyethylene (PE) caps, used as the negative control, were incubated in complete cell culture medium ((Dulbecco’s Modified Eagle Medium (DMEM) supplemented with 10% fetal bovine serum (FBS) and 1% penicillin/streptomycin/amphotericin B (P/S/AmpB)) for 72 h, and the resulting extracts were subsequently used in the test. Complete culture medium alone served as the blank control, and sodium lauryl sulfate (SLS, 0.2 mg/mL in medium) served as the positive control.

### 2.4 Human ACL Explant Model for ES-Lig Infiltration

Human ACL samples were collected from 8 patients undergoing total knee arthroplasty (TKA) at the Nuffield Orthopaedic Centre (Oxford, UK), under informed consent and ethical approval from the TALISMAN OMB project (REC reference: 24/SC/0224). Remnant ACL tissue obtained during TKA was of sufficient size to be wrapped around the ES-Lig scaffold. Patient donors included 4 males and 4 females, with a mean age of 73.1 years (range: 64–82 years). No patients had prior ligament injury or systemic inflammatory disease. Samples were trimmed to a uniform size of approximately 5 mm × 10 mm to match the dimensions of the ES-Lig prior to culture. To assemble the explant model, each ES-Lig scaffold was secured within the ACL tissue using 7–0 sutures (PDS II, Ethicon, Somerville, USA). The ACL/ES-Lig explants were then cultured in complete cell culture medium (DMEM supplemented with 10% FBS and 1% penicillin–streptomycin) at 37 °C, with media changes every two days. After the culture period, samples were transferred to a 24-well plate, and sutures were removed.

Samples were fixed in 10% neutral buffered formalin for 24 h at 4 °C, washed with PBS, and cryoprotected in 30% sucrose for 72 h at 4 °C. Samples were embedded in optimal cutting temperature (OCT) compound, frozen using a dry ice-methanol bath, and stored at -20 °C. Sections (8 μm) were obtained using a Bright OTF5000 Cryostat (Bright Instruments, Huntingdon, UK) and collected onto slides.

### 2.5 Fluorescent Staining and Imaging

Tissue sections were washed with PBS, followed by permeabilisation with 0.2% Triton X-100 (Sigma-Aldrich, St. Louis, MO, USA) in PBS for 1 h. After additional PBS washes, sections were blocked for 1 h in a solution of PBS supplemented with 1% bovine serum albumin (BSA; Sigma-Aldrich, St. Louis, MO, USA) and 0.05% Triton X-100. Alexa Fluor 488 Phalloidin (1:2000 in blocking buffer) was applied for 1 h at 37 °C in a humidity chamber, followed by three PBS washes. Nuclear counterstaining was performed using 4′,6-diamidino-2-phenylindole (DAPI; Thermo Fisher Scientific, Waltham, MA, USA) at 1:1000 in blocking buffer for 5 min, washed again, and mounted with a coverslip. Images were acquired on a Zeiss AXIO Imager M1 Upright Fluorescence Motorised XYZ Microscope (Zeiss, Oberkochen, Germany), using filter sets for Alexa Fluor 488 (FITC channel) and DAPI under a 20 × /0.8 numerical aperture (NA) objective.

### 2.6 Direct Cell Contact Assay and Non-Invasive Microscopy

Green fluorescent protein (GFP)-expressing human mesenchymal stem cells (hMSCs) were generated and cultured in-house following the previously described protocol [32], and seeded into a soft chamber bioreactor. Briefly, cells were maintained in standard growth medium (DMEM/F-12 supplemented with 10% FBS and 1% P/S/AmpB) and seeded into chambers at a density of 5 × 10^5^ cells in 200 μL of medium. Cell viability was evaluated using the PrestoBlue assay (Invitrogen, Paisley, UK) and was conducted on days 0, 1, 2, 6, 10, and 14 according to established protocols. Cell distribution and morphology were assessed on days 0, 1, 2, 6, 10, and 14 through the bioreactor chamber’s walls using confocal laser scanning microscopy at 21 °C with an inverted microscope (LSM 880, Carl Zeiss Microscopy GmbH, Jena, Germany) equipped with an Airyscan detector. GFP-expressing cells were visualised using a 488 nm argon laser. Images were acquired with a pixel size of 1.66 μm, pinhole size of 1 Airy unit, and 8-bit depth. Z-stacks of 20 slices at 0.5 μm intervals were collected, and maximum intensity projections were generated using ImageJ software (National Institutes of Health, Bethesda, MD, USA).

### 2.7 Ex vivo ACLR in Sheep Cadaveric Limbs

ACLR was first performed on sheep cadaveric limbs to ensure reproducibility before in vivo implantation. The limbs were obtained from the Royal Veterinary College (London, UK) following routine euthanasia unrelated to this study. Limbs were retrieved within 6 h post-mortem and stored at − 20 °C until use. The limbs were positioned in a supine orientation, and a midline longitudinal skin incision was made. The patella was laterally dislocated, and a partial excision of the fat pad was performed to expose the ACL, which was carefully transected using a scalpel.

A 2-mm K-wire was inserted at the inferomedial side of the tibial tuberosity and directed toward the centre of the native ACL footprint using an aimer with an outside-in technique, targeting the space between the anteromedial (AM) and posterolateral (PL) bundles. A tibial tunnel was created using a 4.5-mm cannulated drill over the guide wire, followed by a 6-mm cannulated drill. A femoral tunnel was created at the centre of the femoral ACL insertion, employing an inside-out technique. The femoral tunnel was created at the centre of the femoral ACL insertion site using an inside-out technique, also drilled in two steps (4.5 mm followed by 6 mm) to form a 6 mm socket of at least 15 mm in depth. Two types of grafts were prepared: ES-Lig and autograft harvested from the superficial digital flexor tendon (SDFT) using a 5 mm tendon stripper. Grafts were doubled over a continuous loop EndoButton (Smith & Nephew, Watford, UK) to form double-stranded constructs approximately 5 mm in diameter and 65 mm in length. A 2–0 suture (Vicryl, Ethicon, Somerville, USA) was whipstitched on each side of the graft to assist in pulling it through the tunnels. The graft was passed from the tibial to the femoral tunnel using a suture passer, and the EndoButton was flipped and secured on the lateral femoral cortex. Tibial fixation was achieved using a 7 mm × 25 mm interference screw (Smith & Nephew) while maintaining the knee in full extension under 40 N of initial tension. Biomechanical tests were subsequently performed to evaluate the primary stability of the ES-Lig construct before in vivo implantation.

### 2.8 In vivo ACLR in Sheep

All procedures were approved by the Institutional Animal Care and Use Committee (IACUC) of the University of Oxford (Protocol No. P16FAA0A) and conducted at the Royal Veterinary College in accordance with the Animal Research: Reporting of In Vivo Experiments (ARRIVE) 2.0 guidelines. Two healthy adult female Mule sheep (aged approximately 1–2 years and weighing approximately 60–70 kg) were included in a pilot study to evaluate the surgical feasibility, scaffold handling, and preliminary biological response of the ES-Lig scaffold for ACLR. Both animals received the same intervention (ES-Lig scaffold), and no group allocation or blinding was required. Sheep were acclimatised for 7 days and underwent pre-operative veterinary assessment. On Day 0, general anaesthesia was induced using intravenous ketamine and midazolam, and maintained with sevoflurane in oxygen/medical air. Analgesia included fentanyl transdermal patches applied 12 h prior to surgery and replaced 60 h later, supplemented with intraoperative fentanyl infusion and postoperative non-steroidal anti-inflammatory drugs (NSAIDs; meloxicam). Antibiotics were administered from Day 0 for 5 consecutive days. Pain and recovery were monitored at least twice daily using clinical scoring and the Sheep Grimace Scale. Humane endpoints were defined under moderate severity.

Each sheep underwent ACL reconstruction on the right hind limb using the ES-Lig scaffold. The limb was shaved and sterilised in a standard sterile manner while the sheep were positioned supine under general anaesthesia. The surgical procedure, including exposure of the ACL, transection, and preparation of both tibial and femoral tunnels, followed the same steps as the ex vivo cadaveric model. Finally, the incision was closed in layers, starting with the deep fascia of the vastus lateralis muscle. A bulky cotton dressing was applied postoperatively and removed 48 h after surgery. Animals were allowed to recover in sternal recumbency and returned to group housing after 14 days, with ad libitum access to hay, concentrates, and water. Gait was evaluated at pre-surgery, 4 weeks, and 10 weeks post-surgery. At 10 weeks postoperatively, animals were humanely euthanised by intravenous barbiturate overdose followed by confirmation of death. Hind limbs were disarticulated at the midfemur. One limb was processed for biomechanical testing on the day of collection, and the other for histological analysis. All procedures conformed to UK Schedule 1 guidelines and institutional humane endpoint policies.

### 2.9 Biomechanical Testing of Reconstructed Joints

Following either ex vivo reconstruction or in vivo retrieval at 10 weeks postoperatively, sheep hind limbs were processed for mechanical testing. All surrounding muscles were removed while preserving the major stifle ligaments and menisci. The femur and tibia were sectioned approximately 120 mm from the stifle joint line and mounted for mechanical evaluation using a Zwick tensile testing machine (Zwick Roell Group, Ulm, Germany).

#### 2.9.1 Anterior Drawer Test

For the anterior drawer testing, a custom-built testing fixture was utilised to hold the joint at 90° of flexion. After applying a 5 N pre-load, the femur was displaced upward at a rate of 1 mm/s to simulate anterior tibial displacement until a load of 50 N was reached [33]. The anterior–posterior translation, defined as the femoral displacement relative to the tibia under a 50 N load, was recorded.

#### 2.9.2 Tissue Dimension of the Graft

All soft tissues were removed from the knee specimen, preserving only the native ACL or graft. The cross-sectional area of the native ACL or graft was measured at three locations within the intra-articular portion: proximal (near the tibial insertion), mid-substance, and distal (near the femoral insertion) using callipers. Assuming an elliptical geometry, the area at each site was calculated accordingly [34]. The average of the three values was used as the representative cross-sectional area of the native ACL or graft.

#### 2.9.3 Stress Relaxation Test

Joints were subjected to stress relaxation testing using a custom-built fixture that maintained 45° of knee flexion. Each specimen was pre-loaded to 10 N, then strained to 3% strain at a rate of 2 mm/s and held for 20 min. The force decay between 15 and 20 min was recorded to characterise stress relaxation behaviour [35].

#### 2.9.4 Pull-Out Test and Stiffness Measurement

The specimen was preconditioned with a static preload of 5 N for 10 min, followed by 10 cycles of loading and unloading (3% strain) at 20 mm/min. Then, each specimen was loaded to failure at 50 mm/min [36]. Failure modes were recorded. The structural properties of the native ACL or graft were determined from the load-elongation curve. Maximum load and elongation at failure were defined as the load at the point of failure of the specimen. Linear stiffness was defined by the slope of the load-elongation curve, which was determined by applying a least-squares linear regression analysis to the data curve between the endpoint of the toe region and the point that starts to bend before failure.

### 2.10 Magnetic Resonance Imaging Assessment

Post-mortem magnetic resonance imaging (MRI) scans were conducted before dissection, with hind limbs harvested and scanned within 60 min. Imaging was carried out using a 3.0 T Siemens Magnetom Vida MRI scanner (Siemens Healthineers, Erlangen, Germany) equipped with the syngo MR XA60 software and an Ultra Flex Large coil. The stifle joints were positioned in lateral recumbency with 45° flexion. MRI sequences included proton density (PD) and fat-saturated proton density (PD-FS) imaging with typical parameters as follows: slice thickness 2 mm, TR/TE ranging from 3360–7490 ms/31–39 ms, base resolution 448–576, and GRAPPA acceleration factor 2. T1-weighted sequences included high-resolution sagittal T1 SPACE (slice thickness 0.6 mm, TR/TE 600/28 ms, CAIPIRINHA acceleration factor 4) and T1 VIBE WE (slice thickness 0.6 mm, TR/TE 10.5/4.92 ms, base resolution 320, GRAPPA factor 2). Data analysis was performed using the RadiAnt DICOM viewer (version 2025.1, Medixant, Poznań, Poland).

### 2.11 Computed Tomography Assessment

Computed tomography (CT) scans were conducted using a Canon scanner (Aquilion One Genesis Edition, software version 8.3SP9000). Scanning parameters were as follows: tube voltage 120 kV, tube current 150 mA, pitch HP 0.938 / PF 0.75, and acquisition slice thickness 1.25 mm. For high-resolution bone analysis, images were reconstructed with a slice thickness of 0.5 mm and a slice interval of 0.25 mm. CT data were then analysed using RadiAnt DICOM viewer. Measurements of the femoral bone tunnel diameter at the aperture, middle, and exit were performed on circular planes perpendicular to the long axis of the femoral tunnel axis. Tunnel diameters were calculated as the average of two orthogonal diameters (D_1_ and D_2_), representing the long and short axes of the elliptical cross-section. Tunnel diameter = (D_1_ + D_2_) / 2 (Fig.S7).

### 2.12 Three-Dimensional (3D) Reconstruction of ES-Lig and Fixation Structures

To generate a comprehensive 3D visualisation of the reconstructed joint, imaging datasets were processed using Materialise’s Interactive Medical Image Control System software (Mimics, version 21.0; Materialise, Leuven, Belgium) [37]. The femoral and tibial bone tunnels, interface screw, and EndoButton fixation device were segmented from CT datasets, whereas the ES-Lig graft was reconstructed based on MRI scans. Individual anatomical structures were segmented manually using thresholding, mask editing, and region-growing algorithms in Mimics. The segmentation accuracy was confirmed by consensus between a musculoskeletal surgeon (G.B.) and an orthopaedic sports medicine fellow (J.L.). The segmented surfaces were then exported as stereolithography (STL) files and imported into Geomagic Wrap 2021 (3D Systems, Rock Hill, SC, USA) for mesh refinement, smoothing, and alignment.

### 2.13 Paragon Staining

Bone tunnel histological analysis was performed using Paragon staining to evaluate ES-Lig integration at the graft-bone interface. Tissue specimens were first fixed in 10% neutral buffered formalin for 48 h, then rinsed in PBS. Samples were dehydrated in a graded ethanol series (70%, 80%, 90%, 95%, and 100%), followed by infiltration and embedding in methyl methacrylate (Technovit® 9100, Kulzer GmbH, Hanau, Germany). Embedded blocks were sectioned to a thickness of 40 μm using a precision diamond saw (Exakt 300 CP, EXAKT Advanced Technologies GmbH, Norderstedt, Germany), and further ground and polished to a final thickness of 20 μm using an Exakt micro-grinding system (EXAKT 400CS, EXAKT Advanced Technologies GmbH). Sections were stained with Paragon stain (Paragon C&T, Alizarin red and methylene blue based, Polysciences Inc., Warrington, PA, USA) to visualise bone, soft tissue, and ES-Lig interface structures. High-resolution digital images were acquired using bright-field microscopy (Axio Imager M1, Carl Zeiss Microscopy GmbH, Jena, Germany). Microvessel density was quantified as vessels per mm^2^ using ImageJ on three non-consecutive sections per specimen. Analyses were performed within predefined regions of interest covering the graft and bone-tunnel interface. Vessels were counted only when a visible lumen was present to exclude clefts and artefacts.

### 2.14 Haematoxylin & Eosin (H&E) Staining

Frozen tissue sections (8 μm thickness), prepared from OCT-embedded samples using a Bright OTF5000 cryostat (Bright Instruments, Huntingdon, UK), were mounted onto glass slides and air-dried. Sections were stained with Harris’ haematoxylin solution (Sigma-Aldrich, St. Louis, MO, USA) for 60 s, rinsed in distilled water for 10 s, and treated with 5 N ammonium hydroxide (Merck, Darmstadt, Germany) for 10 s to enhance nuclear contrast. Sections were rinsed again in distilled water before counterstaining with eosin Y (Sigma-Aldrich, St. Louis, MO, USA) for 5 s. Dehydration was performed through a graded ethanol series (95% and 100%), followed by clearing in xylene (Fisher Chemical, Hampton, NH, USA) for two cycles of 10 s each. Slides were air-dried for 5 min and mounted with dibutylphthalate polystyrene xylene (DPX; Sigma-Aldrich, St. Louis, MO, USA) using 1500 μL per slide, then sealed with glass coverslips. Bright-field imaging was conducted using a Zeiss Axio Imager M1 microscope (Carl Zeiss Microscopy GmbH, Jena, Germany) equipped with a pE-300 white LED illumination system (CoolLED Ltd., Andover, UK). Images were acquired at magnifications ranging from 4 × to 100 ×. Cell density was measured as nuclei per mm^2^ using ImageJ on three non-consecutive sections per specimen.

### 2.15 Statistical Analysis

All statistical analyses were performed using GraphPad Prism version 9.5.1 (GraphPad Software, San Diego, CA, USA). Data are presented as mean ± standard deviation (SD), unless otherwise stated. Normality of distribution was assessed using the Shapiro–Wilk test. For comparisons of biomechanical properties (e.g. stiffness, pull-out strength, tensile stress) between three groups (ES-Lig, autograft, and native ACL), a one-way analysis of variance (ANOVA) followed by Tukey’s multiple comparisons test was used when data were normally distributed. To analyse cellular infiltration into ES-Lig across three timepoints (weeks 0, 2, and 4) in the ACL explant model, data were analysed using the Kruskal–Wallis test followed by Dunn’s correction for multiple comparisons. For the longitudinal cell viability assay in the bioreactor, involving repeated measurements over six timepoints (days 0, 1, 2, 6, 10, and 14), a repeated measures one-way ANOVA with Geisser-Greenhouse correction and Holm-Šidák’s multiple comparisons test was used. A two-tailed *p* value of < 0.05 was considered statistically significant.

## 3 Results and Discussion

### 3.1 Assembly and Design of ES-Lig Inspired by Native ACL

**Fig. 1:**
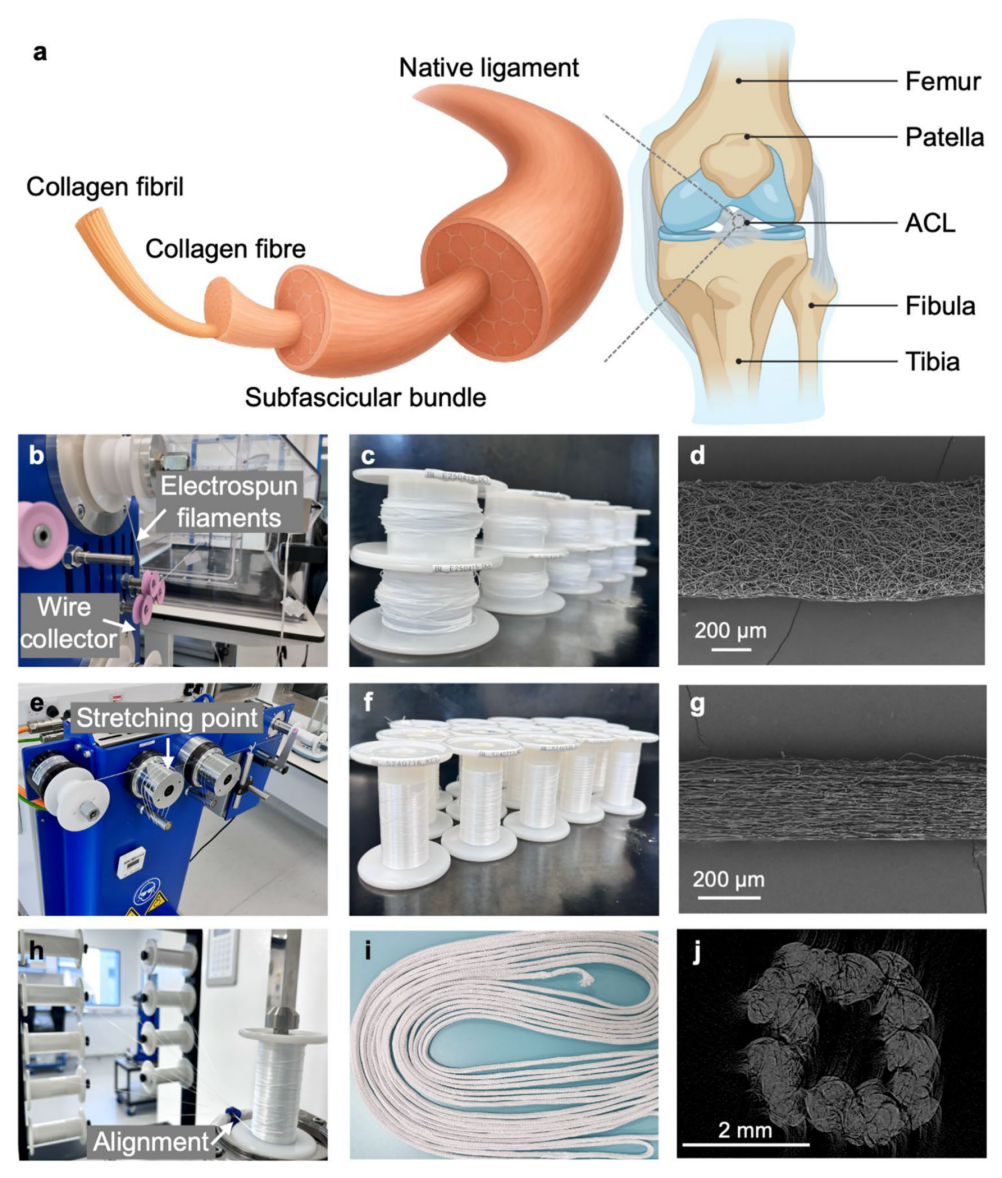
Structure of a native ACL and Manufacturing of ES-Lig. **a** Schematic of a native ACL structure. **b–d** Electrospinning in ES-Lig manufacturing: automated wire-collection electrospinning set-up (the upper arrow shows the automated peeling off the spun filaments, and the lower arrow shows the automated wire collection) (**b**), scaledup electrospun filaments (**c**), and SEM image of electrospun filaments. Scale bar 200 µm (**d**). **e–g** Stretching in ES-Lig manufacturing: drawing machine set-up for stretching (the arrow shows the stretching point happens due to the different ratios of the cylinders) (**e**), scaledup stretched filaments (**f**), and SEM image of stretched filaments. Scale bar 200 µm (**g**). **h** Multifilament alignment assembly (the arrow shows the alignment point where 9 stretched filaments were combined into one aligned bundle). **i** Scaled-up braided cord as large scale of ES-Lig. **j** MicroCT imaging of the cross-section of ES-Lig

Native ACLs are a critical load-bearing structure within the knee joint, composed of organised ECM of collagen fibrils, collagen fibres, and subfascicular bundles (Fig. 1a) [1, 38]. While autografts are often used for ACLR in high incidence of ACL injuries, their limitations, including donor site morbidity, limited availability, and inconsistent outcomes, highlight the urgent need for artificial ligament substitutes. To address this clinical demand, we designed a fully degradable and biomimetic ES-Lig, which is inspired by the native ACL’s ECM and optimised to balance gradual scaffold degradation and sustained mechanical support during tissue regeneration. A large-scale electrospinning process was employed in an ISO Class 7 Cleanroom to produce continuous filaments, a critical step towards upscaling fabrication for clinically relevant applications. The electrospun filaments were successfully collected onto wire collectors, yielding spools of fibrous material through automated separation of the spun filaments from the wire (Fig. 1b, c). SEM imaging revealed a highly porous, randomly oriented fibrous structure within the filaments (Fig. 1d), which is expected to promote cell infiltration but with relatively low mechanical strength in the as-spun state. To enhance the mechanical properties and further align the internal fibre structure, the electrospun filaments underwent a stretching process under the different ratios of the two cylinders (Fig. 1e). Post-stretching, the filaments exhibited increased tensile strength and reduced diameter, as observed both macroscopically (Fig. 1f). This was confirmed through SEM imaging of stretch filaments (Fig. 1g). This showed the stretched filaments with a more compact and aligned morphology compared to the as-spun state, which is expected to improve load-bearing capacity while still maintaining an open structure to facilitate tissue integration.

For the fabrication of ligament-like constructs, multiple stretched filaments (n = 9) were aligned into bundles without twisting (Fig. 1h). This alignment method was intentionally selected over traditional twisting techniques to enhance cellular infiltration throughout the construct, facilitating uniform tissue regeneration. The bundled filaments were then braided into long cords (approximately 8 m in length) to produce scalable ES-Lig constructs (Fig. 1i). This scale-up capability is crucial and tuneable, enabling the production of approximately 25 ES-Ligs from this 8-m single braided cord, thus meeting the demands for both experimental and potential clinical applications. The whole manufacturing procedures were standardised for the production of ES-Lig in a functionalised cleanroom as a medical device (Fig. S1). MicroCT imaging of the ES-Lig cross-section (Fig. 1j) revealed a biomimetic architecture with a hollow structure. It is expected to not only provide initial mechanical support but also promote tissue infiltration and neo-tissue formation, essential for long-term graft remodelling and integration. Through fibre stretching, filament alignment, and controlled braiding, our approach successfully produced a device that mimics key features of the ECM structure of native ligament, providing good mechanical strength and maintaining porosity and space for tissue integration. This biomimetic design can establish a scalable platform for bioengineered ligament production.

### 3.2 Characterisation and Degradation Study of ES-Lig

**Fig. 2:**
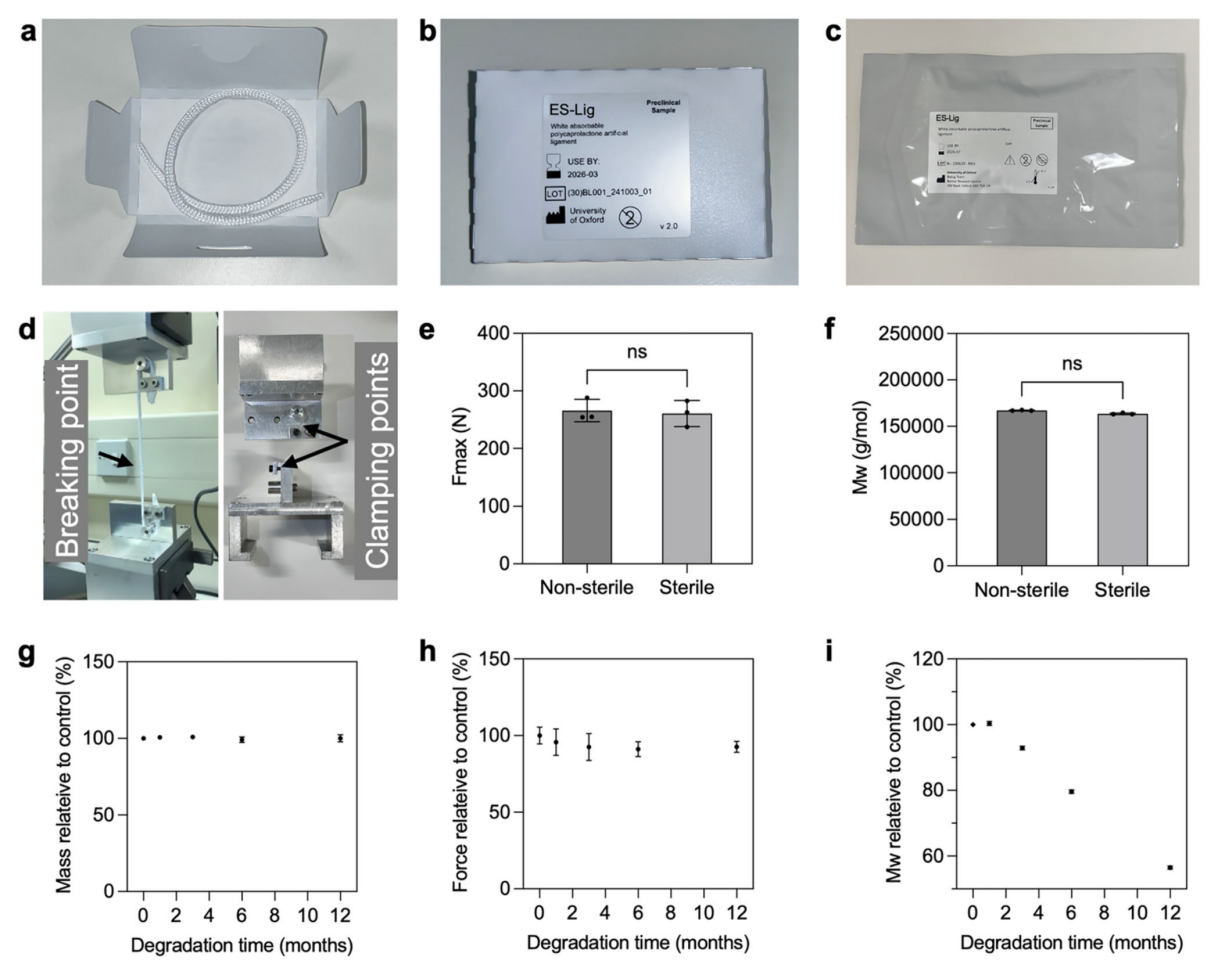
Characterisation and degradation of ES-Lig. **a-c** Labelling and packaging of ES-Lig, including the die card insert (**a**), labelled die card inserts (**b**), and outer packaging with sealed ES-Lig product (**c**). **d** Tensile test setup with tailored clamps for ES-Lig and filament mechanical property measurement. **e** Breaking force of ES-Lig before and after EtO Sterilisation. **f** Molecular weight measurement by GPC of ES-Lig before and after EtO Sterilisation. **g–i** Degradation studies of ES-Lig over 12 months: mass measurement (**g**), breaking force (**h**), and molecular weight (**i**) during the 12-month degradation period

ES-Lig, the bioresorbable ligament scaffold, was developed with both structural integrity, biomechanical functions and tissue regeneration (Fig. 1 and Fig. S1). The physical product labelling and packaging (Fig. 2a–c) were designed and conducted in a cleanroom to maintain sterility and usability in clinical environments. The final product was delivered in a sealed, labelled pouch containing a die card insert with the ES-Lig braided cord properly coiled. To evaluate its mechanical performance, ES-Lig underwent tensile testing using a custom clamping setup tailored to the filament form (Fig. 2d). This setup allowed for consistent and accurate measurement of mechanical properties under tension. Initial testing compared the breaking force of ES-Lig pre- and post-EtO sterilisation. No statistically significant difference was observed in breaking force between the non-sterile and sterile samples with only 1.89% drop, suggesting the sterilisation process does not compromise mechanical integrity (Fig. 2e). However, gel permeation chromatography (GPC) revealed a slight (2.19%) decrease in molecular weight after sterilisation (Fig. 2f). Despite this reduction, the polymer chains retained sufficient length to ensure the mechanical functionality of ES-Lig remained within clinically acceptable limits.

The degradation behaviour of ES-Lig is an essential aspect to investigate alongside the tissue regeneration. The degradation profile was assessed over 12 months by measuring mass retention, breaking force, and molecular weight (Fig. 2g–i). Mass loss was negligible during this period, with less than a 1% reduction (Fig. 2g). Notably, despite this minimal mass loss, the breaking force remained stable, showing no statistically significant difference compared to baseline (month 0), with reductions of 4.33%, 7.50%, 8.83%, and 7.33% at months 1, 2, 6, and 12, respectively (Fig. 2h).

Although the reduction ratio at 12 months appeared slightly lower than at 6 months, this difference was not statistically significant and is most likely attributable to natural variation in testing. This degradation is supported by the ongoing molecular weight decline (Fig. 2i) without structural reorganisation in SEM imaging (Fig. S2 and S3). Together, these findings indicate that ES-Lig degrades predominantly through bulk chain scission rather than structural reinforcement, while retaining sufficient mechanical integrity throughout the first year of degradation for supporting tissue healing. This degradation profile is consistent with the literature on PCL [39], where bulk chain scission proceeds in vitro and in vivo, confirming the expected progressive loss of molecular weight and mechanical properties under physiological conditions. A pronounced drop in molecular weight was observed from month 3 onwards, with an initial 7.13% reduction (Fig. 2i), confirming ongoing polymer chain scission and molecular breakdown. By 12 months, molecular weight had decreased significantly (43.5%), a linear trend expected to continue until complete resorption around 27 months, aligning with the timeline for native tissue integration and functional maturation. SEM imaging was performed to observe morphological changes in both stretch filaments and braided ES-Lig structures over time (Fig. S2 and S3). In the stretched filaments (Fig. S2), surface erosion was not obvious over 12 months, but in the braided ES-Lig (Fig. S3), demonstrated more complex degradation patterns due to its higher surface area, with noticeable fraying and strand separation progressing over time. Despite these morphological changes, the structural framework remained sufficiently intact to support tissue regeneration during early healing stages. ES-Lig demonstrates key properties for ligament regeneration, maintaining mechanical strength post-sterilisation and degrading in a controlled manner to support tissue healing for at least one year. Full degradation is expected to occur over approximately 4 years, by which time the regenerated ligament should have assumed its full load-bearing role.

### 3.3 ES-Lig Exhibits Mechanical Stability Comparable to Autografts Ex vivo

**Fig. 3:**
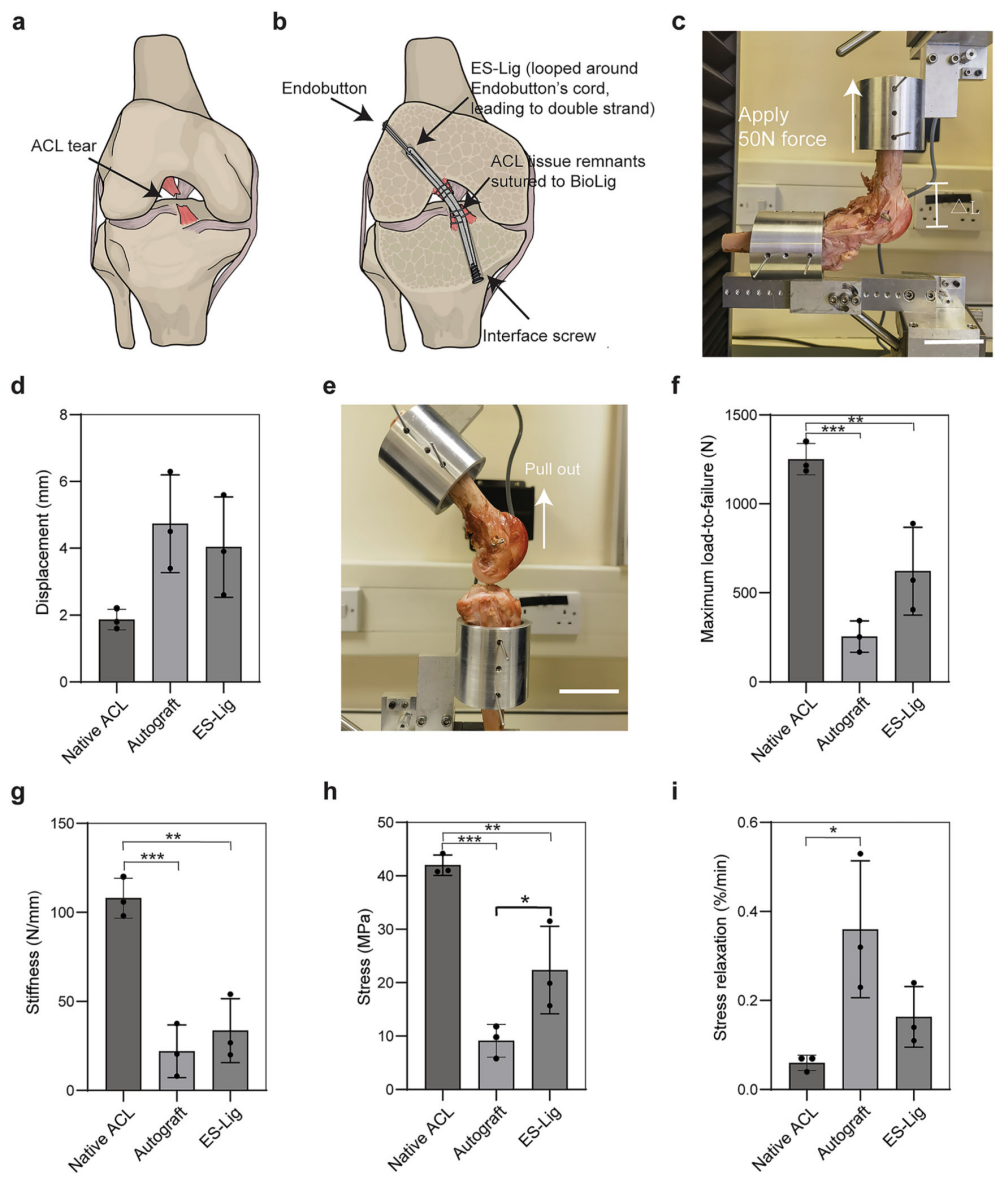
Mechanical properties of ES-Lig ACLR fixation and controls. **a, b** Schematic illustration of an ACL tear (**a**) and ES-Lig fixation method (**b**), showing the interface screw on the tibial side and Endobutton on the femoral side. ES-Lig is looped around the Endobutton’s cord, forming a double-strand structure, with ACL tissue remnants sutured to ES-Lig. **c** Experimental setup for the anterior drawer test, evaluating joint stability by measuring femoral-to-tibial displacement (△L). Scale bar, 5 cm. **d** Experimental setup for pullout test, assessing maximum loading-to-failure by pulling the grafts out of the bone tunnel. Scale bar, 5 cm. **e** Anterior drawer test results. **f** Pull-out strength. **g** Stiffness measurements. **h** Stress. **i** Stress relaxation test. Statistical significance was denoted as follows: ***p*** < 0.05 (*), ***p*** < 0.01 (**), *p* < 0.001 (***)

To evaluate the mechanical performance of ES-Lig in ACLR, a series of biomechanical tests were conducted to compare its stability and load-bearing properties with those of native ACL and autograft tissue (Fig. 3). A standard fixation configuration was used in all reconstructions, using an Endobutton loop at the femoral side and an interference screw at the tibial side (Fig. S4) [40]. Both ES-Lig and SDFT autografts were doubled over the Endobutton loop to form a double-strand construct (Fig. 3a, b).

Anterior drawer testing was performed by applying a 50 N anterior force to the tibia while the femur was held fixed, simulating anterior–posterior joint instability (Fig. 3c). The displacement measured for the ES-Lig group (4.0 ± 1.2) mm was comparable to autografts (4.7 ± 1.2) mm and slightly greater than that of native ACL (1.9 ± 0.2) mm (Fig. 3d). This level of joint stability is essential for preventing anterior tibial translation during locomotion [41] and falls within the functional range reported for successful ACLR in ovine models using either autografts [33, 42–46] or artificial grafts [47].

Fixation strength was evaluated using the pull-out test, which assessed the maximum force required to pull the graft out of the bone tunnel (Fig. 3e). The native ACL demonstrated significantly greater strength (1250.9 ± 72.0) N than both ES-Lig (621.5 ± 201.1) N and autografts (255.2 ± 72.2) N (Fig. 3f). Failure consistently occurred at the tibial fixation site rather than within the graft, indicating sufficient intrinsic strength of the scaffold itself. Notably, this force exceeds the minimum threshold (150 N) required to resist physiological knee loads in ovine models [48], suggesting that ES-Lig can achieve stable early fixation without risk of graft slippage.

The measured stiffness of ES-Lig ((33.6 ± 14.6) N/mm) closely matched that of autografts ((22.0 ± 12.1) N/mm) while remaining substantially lower than the native ACL ((108.0 ± 9.1) N/mm) (Fig. 3g). This moderate stiffness is unlikely to induce stress shielding, which is typically associated with constructs of markedly higher stiffness than native tissue, and may instead help reduce stress concentrations at the fixation interface during the early postoperative period when soft tissue integration is incomplete [49]. Tensile strength analysis showed that ES-Lig (22.4 ± 6.7) MPa was significantly higher than autografts (9.1 ± 2.5) MPa but lower than native ACL (42.0 ± 1.5) MPa (Fig. 3h). Previous ovine ACLR studies have reported that insufficient early tensile strength (< 10 MPa) increases the risk of elongation and loss of stability [33], whereas mechanical profiles within an intermediate range can maintain joint stability while allowing physiological strain to stimulate collagen remodelling during ligamentisation [35].

Viscoelastic properties were evaluated using a stress relaxation test, which tracks the rate of internal stress decay under constant strain. The native ACL exhibited the slowest relaxation (0.06 ± 0.02) %/min, significantly lower than autografts (0.36 ± 0.12) %/min, while ES-Lig (0.16 ± 0.05) %/min showed no significant difference from either group (Fig. 3i).

### 3.4 ES-Lig Supports Human Cell Viability and Infiltration In Vitro

**Fig. 4:**
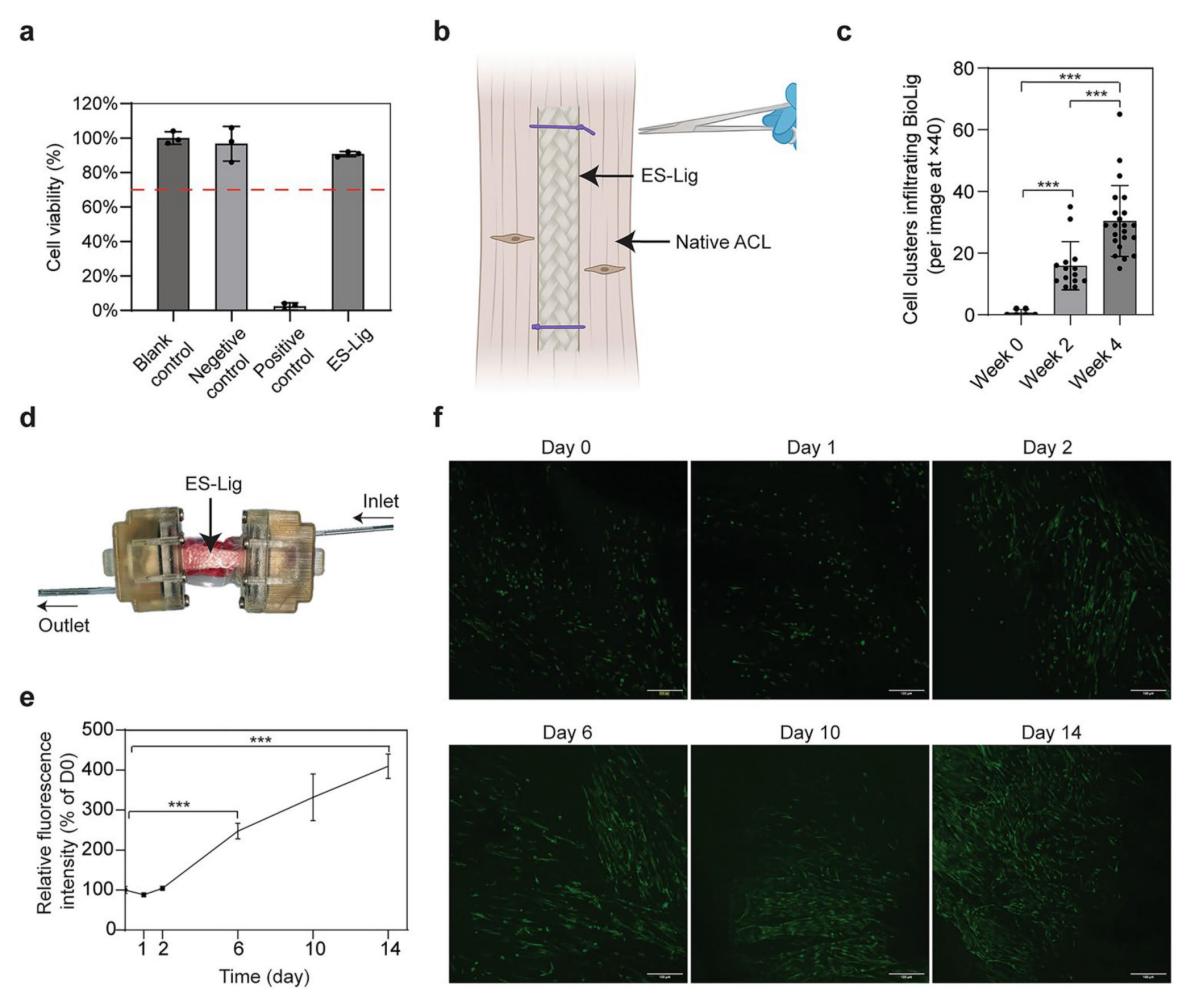
Biocompatibility and cellular infiltration of ES-Lig in vitro. **a** Cytotoxicity of ES-Lig evaluated by the NRU assay. The red dashed line indicates 70% cell viability, the standard threshold for cytotoxicity according to ISO 10993–5. **b** Schematic of the patient-derived ACL explant model used to assess scaffold infiltration. **c** Quantification of cell clusters infiltrating ES-Lig (per image at × 40 magnification), demonstrating progressive cellular infiltration over a 4-week culture period. **d** Integration of ES-Lig within a soft bioreactor system, designed to provide a controlled dynamic culture environment. **e** Viability of hMSCs cultured in the same bioreactor chamber over time, assessed by the PrestoBlue assay. **f** Confocal imaging of hMSCs on ES-Lig under non-invasive observation, showing cellular attachment and aligned orientation along the scaffold surface. Statistical significance was denoted as follows: *p* < 0.001 (***)

Cytotoxicity testing based on the NRU assay, in accordance with ISO 10993-5:2009, confirmed that ES-Lig was non-toxic, indicating its safety for implantation (Fig. 4a). The ES-Lig was secured with the ACL explant tissue using sutures to evaluate cellular infiltration over 4 weeks (Fig. 4b). Quantitative analysis showed a significant increase in cell clusters infiltrating ES-Lig at 2 weeks (15.9 ± 7.5) per image at × 40 and 4 weeks (30.4 ± 11.5) per image at × 40 compared to the initial time point (0.7 ± 1.0) per image at × 40, with a further increase observed between 2 and 4 weeks (*p* < 0.001) (Fig. 4c).

ES-Lig was integrated into a soft chamber bioreactor to examine its support for sustained cell viability in a controlled 3D culture environment (Fig. 4d). hMSCs' viability on ES-Lig was assessed using the PrestoBlue assay over 14 days (Fig. 4e). hMSCs remained metabolically active, with a significant increase in viability observed at day 6 (247.4 ± 19.8) % and day 14 (409.9 ± 30.5) % relative to day 0 (*p* < 0.01). Confocal microscopy further confirmed hMSCs' adhesion and alignment on the scaffold, forming an organised cellular network over time (Fig. 4f). Although mechanical loading was not applied in this study, the bioreactor system is compatible with a humanoid robotic system, enabling physiologically relevant mechanical stimulation in future applications [32].

### 3.5 ES-Lig Integrates With Native Tissue and Restores Function In Vivo

**Fig. 5:**
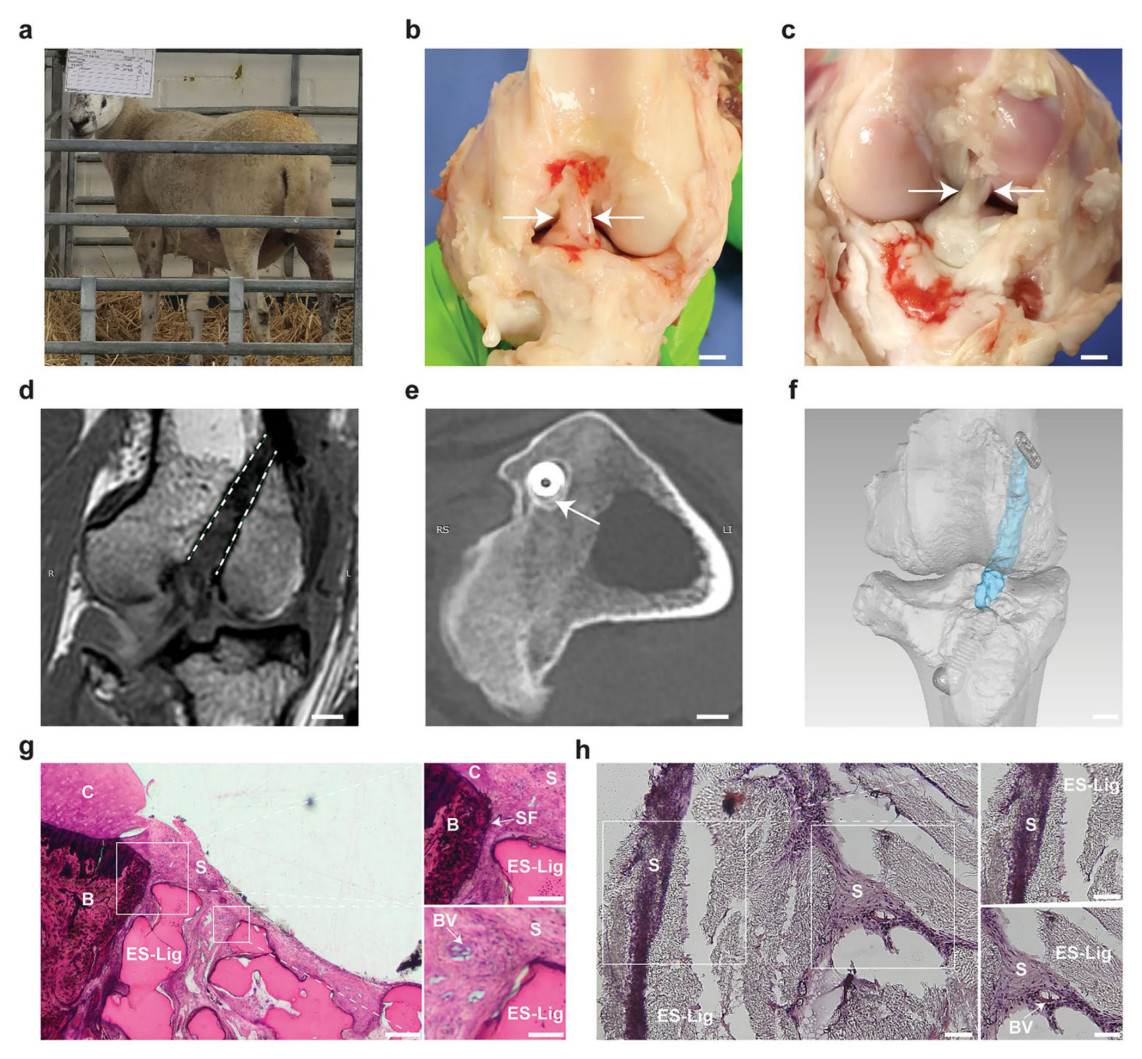
Integration and structural performance of ES-Lig in vivo. **a** Immediate post-operative standing behaviour, indicating tolerance of load-bearing and absence of early motor deficit. **b** Native ACL from the contralateral hindlimb for gross morphological comparison. Scale bar, 5 mm. **c** ES-Lig implanted ACLR in the right hindlimb, showing reconstructed graft in situ with appropriate tension and orientation, as well as visible synovial coverage and surface neovascularisation. Scale bar, 5 mm. **d** Coronal MRI of ES-Lig illustrating graft continuity in the femoral bone tunnel; ES-Lig is indicated by a white dashed outline. Scale bar, 5 mm. **e** Axial CT imaging of the tibial tunnel revealing new bone formation (white arrow) around the interface screw and ES-Lig interface. Scale bar, 5 mm. **f** 3D reconstruction of CT and MRI data, confirming the spatial positioning of ES-Lig (blue) within the joint cavity; femoral and tibial fixation achieved via Endobutton and interference screw, respectively. Scale bar, 5 mm. **g** Paragon staining, showing ES-Lig graft-bone integration within bone tunnel. Scale bar, 200 μm. Top, magnified view reveals Sharpey’s fibre analogues anchoring the ES-Lig to newly formed bone. Scale bar, 100 μm. Bottom, synovial tissue infiltration into ES-Lig with abundant neovascularisation. Scale bar, 50 μm. Labels: B, bone; C, cartilage; S, synovial tissue; BV, blood vessels; SF, Sharpey’s fibres. **h** H&E staining of the mid-substance of ES-Lig, demonstrating cellular infiltration and tissue ingrowth within ES-Lig. Scale bar, 50 μm. Top, surface encapsulation of the graft with host-derived cells. Scale bar, 20 μm. Bottom, synovial tissue ingrowth into the ES-Lig with observable neovascular features. Scale bar, 25 μm. Labels: S, synovial tissue; BV, blood vessels

Evaluation of in vivo integration and mechanical performance in large animal models is crucial for translating ES-Lig into human clinical applications. As a pilot feasibility study, only two sheep were used without an autograft control, following the 3Rs (Replacement, Reduction, Refinement) to minimise animal use. The aim was to assess surgical handling, early host response, and proof-of-concept performance, informing ongoing large-scale GLP (Good Laboratory Practice)-compliant ovine studies with autograft controls and longer follow-up. To this end, ES-Lig was implanted in an ovine ACLR model (Fig. S5). A total of two sheep underwent the procedure; all recovered without complications from anaesthesia. As shown in Fig. 5a, immediate postoperative weight-bearing and autonomous standing were observed, indicative of early tolerance to axial loading. At the 10-week study endpoint, one sheep was used for histological evaluation and the other for mechanical testing. The 10-week time point was selected based on prior ovine ACL reconstruction studies identifying this period as the early ligamentisation phase suitable for assessing graft integrity, fixation stability, and initial host integration [33, 45].

At 10 weeks post-implantation, gross inspection confirmed that the intra-articular portion of the ES-Lig graft maintained structural integrity, with sufficient thickness and tension and no signs of elongation (Fig. 5b). No macroscopic meniscus lesions or cartilage degeneration were observed at the femoral condyles or tibial plateau. The graft was fully covered by synovial tissue, with visible neovas-cularisation on its surface, suggesting a pro-regenerative host response. The femoral and tibial bone tunnel apertures appeared fully sealed. Morphologically, the reconstructed graft closely resembled the native ACL in terms of anatomical positioning, fibre orientation, and continuity within the joint cavity (Fig. 5c). PCL-based materials have previously been reported to exhibit favourable biocompatibility with minimal inflammatory responses in vivo [27]. Consistent with this, no overt inflammatory changes such as joint effusion or peri-graft tissue hyperaemia were observed in this ovine model. Although this suggests good local tolerance, future studies will incorporate quantitative analysis of joint aspirates for pro-inflammatory cytokines such as IL-1β and TNF-α to assess potential subclinical immune responses to PCL degradation byproducts.

MRI demonstrated a continuous low-signal-intensity structure spanning from the femoral to tibial footprints, consistent with intact graft morphology and successful placement within the bone tunnels (Fig. 5d, Fig. S6). Mild joint effusion and synovial membrane hyperplasia were observed, as is commonly observed during the early remodelling phase. The signal intensity at the graft-bone interface indicated early-stage graft integration, which is typical of early-stage integration and enthesis formation [50]. CT imaging further revealed that the femoral bone tunnel remained unchanged (6.2 ± 0.2) mm compared to the initial 6 mm drill size. On the tibial side, the ES-Lig graft and interference screw were surrounded by mineralised tissue, suggesting new bone formation along the interface (Fig. 5e, Fig. S7). Three-dimensional reconstruction of MRI and CT data confirmed accurate spatial positioning of the ES-Lig graft and revealed secure anchoring via femoral Endobutton and tibial interference screw (Fig. 5f).

Paragon staining revealed substantial bone deposition adjacent to the ES-Lig scaffold (Fig. 5g). In the high-magnification region, Sharpey’s fibre analogues were observed bridging the graft and newly formed bone, further supporting mechanical integration. The scaffold was also infiltrated by synovial tissue, with visible neovascular structures (223.1 ± 78.1 vessels/mm^2^) penetrating the porous scaffold architecture. In parallel, H&E staining of the midsubstance of ES-Lig showed extensive cellular infiltration (2091.1 ± 547.5 cells/mm^2^) (Fig. 5h). Host-derived fibro-blast-like cells were observed on the graft surface, while the interior displayed organised matrix deposition and newly formed microvasculature, suggesting active remodelling and early-stage ligamentisation.

At the functional level, the sheep treated with ES-Lig grafts exhibited mild limping at 4 weeks, but by 10 weeks, both were able to bear weight, stand, and walk without visible lameness (Supplementary Movie 1), demonstrating functional recovery post-reconstruction.

### 3.6 ES-Lig Provides Mechanical Stability and Fixation Strength In vivo

The biomechanical performance of ES-Lig in an ovine ACLR model was assessed 10 weeks post-implantation (Table 1). Consistent with ex vivo results, cortical suspensory fixation using an Endobutton at the femoral side (Fig. S8a) and an interface screw at the tibial side (Fig. S8b) remained mechanically intact. Anterior drawer testing showed a mean anterior translation of 1.4 mm, comparable to native ACL (1.6 mm) and lower than reported values for autograft and artificial ligament reconstructions (Table 1). This indicates that ES-Lig restored anterior–posterior joint stability under physiological loading. Stress relaxation test indicated a stress decay rate of 0.16%/min for ES-Lig, which was higher than that of the native ACL (0.04%/min) but remained within an acceptable range for ligament substitutes (Table 1). The pull-out test revealed a maximum force of 376 N, which surpasses the minimum threshold for ovine ACL reconstruction (150 N) and falls within the range of autografts. Although lower than the native ACL (1185 N), the measured strength exceeded that of many artificial ligaments (Table 1). Failure occurred at the tibial fixation site due to graft pull-out (Fig. S8c), suggesting sufficient in-graft strength, with that bone-graft interface being the limiting factor. ES-Lig exhibited a stiffness of 22.8 N/mm, lower than that of native ACL (33.3 N/mm) but within the acceptable range for ovine ACLR models (Table 1). The tensile strength of ES-Lig was measured at 11.8 MPa, lower than the native ACL (41.0 MPa), but above reported values for autograft (6.3–10.0 MPa) (Table 1). Together, these findings demonstrate that ES-Lig provides sufficient mechanical stability and fixation strength in the large animal ACLR model to support early joint function and tissue integration.

## 4 Conclusions

In this study, we developed ES-Lig, a fully degradable, braided scaffold composed of PCL electrospun yarns, designed to replicate the ECM architecture and biomechanical function of the native ACL. A scalable manufacturing workflow was established, integrating electro-spinning, stretching, filament alignment, and braiding under cleanroom conditions. ES-Lig achieves a balanced combination of mechanical robustness, controlled biodegradation, and biological responsiveness, supporting early tissue repair and functional recovery. Ex vivo testing demonstrated mechanical performance comparable to autografts, while in vivo results in a sheep ACL reconstruction model confirmed early restoration of joint function. Unlike non-degradable synthetic grafts (e.g., LARS, Dacron), which exhibit poor long-term integration, or natural collagen-based scaffolds that degrade too rapidly and lack strength, ES-Lig offers a resorbable, mechanically competent alternative optimised for regenerative ligament repair. Currently, large-scale GLP-compliant ovine studies are underway to assess long-term degradation, graft durability, and joint preservation. These include autograft control groups, 52-week follow-up, and expanded histological, biomechanical, and imaging assessments. In addition, parallel in vitro degradation studies are ongoing to complement the in vivo data and provide insights into the long-term behaviour of the scaffold. These preclinical data will be critical for regulatory submissions and the design of future first-in-human clinical trials. Future work will also focus on elucidating the molecular biological mechanisms by which BioLig scaffolds modulate cell behaviour and ligament regeneration. Taken together, these findings position ES-Lig as a clinically translatable, next-generation ligament substitute that not only meets the mechanical demands of ACL reconstruction but also lays the foundation for a new class of regenerative implants designed to accelerate rehabilitation, restore active lifestyles, and redefine the future of soft tissue repair.

## Supplementary Material

The online version contains supplementary material available at https://doi.org/10.1007/s42765-025-00632-8.

Supplementary file

## Figures and Tables

**Table 1:** Biomechanical comparison of ES-Lig, autograft and native ACL following 10-week implantation in an ovine model *

	Anterior drawer test displacement (mm)	Pull-out force (N)	Stiffness (N/mm)	Tensile strength (MPa)	Stress relaxation test (%/min)	References
ES-Lig	1.4	376	22.8	11.8	0.16	-
Native ACL	1.6	1185	33.3	41.0	0.04	-
Native ACL (reported)	0.6–2.8	759–1854	59–266.4	41.9–87.9	NR	[1–9]
Autograft^#^ (reported)	2.9–9.3	211–599	33.5–152.4	6.3–10.0	NR	[1–7, 10–13]
Artificial graft^#^ (reported)	2.8	42–836	9–147	NR	NR	[9, 14, 15]

* For reference, values from the literature have been included

# Values reported are either at week 10 or week 12, with fixation using an EndoButton (femoral side) and interference screw (tibial side)

*ACL* Anterior cruciate ligament, *NR* Not reported

## Data Availability

The data that support the findings of this study are available from the corresponding author upon reasonable request.
