# Supplementary material for: Translational Potential of an Electrospun Polycaprolactone Scaffold for Anterior Cruciate Ligament Reconstruction
Source: Adv Fiber Mater. 2025 Nov 3;8(2):560–76. doi: 10.1007/s42765-025-00632-8 (PMC7618662; doi:10.1007/s42765-025-00632-8)
Supplement: Supplementary file 1 — Supplementary file1 (DOCX 7927 KB) [file 42765_2025_632_MOESM1_ESM.docx]

**Supplementary Material**


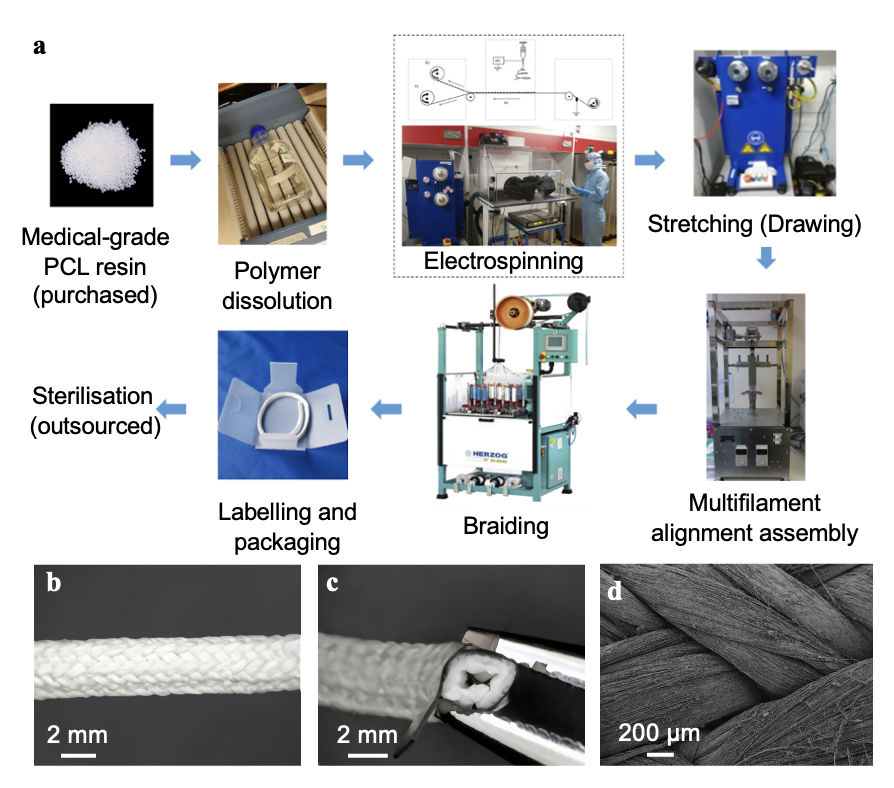


**Fig. S1 Standardised manufacturing of ES-Lig in an operational cleanroom and characterisation of ES-Lig. a,** Schematic overview of the ES-Lig manufacturing process, including PCL polymer dissolution, electrospinning, stretching, multifilament alignment, braiding, labelling and packaging, followed by outsourced sterilisation. **b-c,** Photographs of ES-Lig showing the braided surface structure and cross-sectional view. Scale bar: 2 mm. **d,** SEM image of ES-Lig. Scale bar: 200 µm.


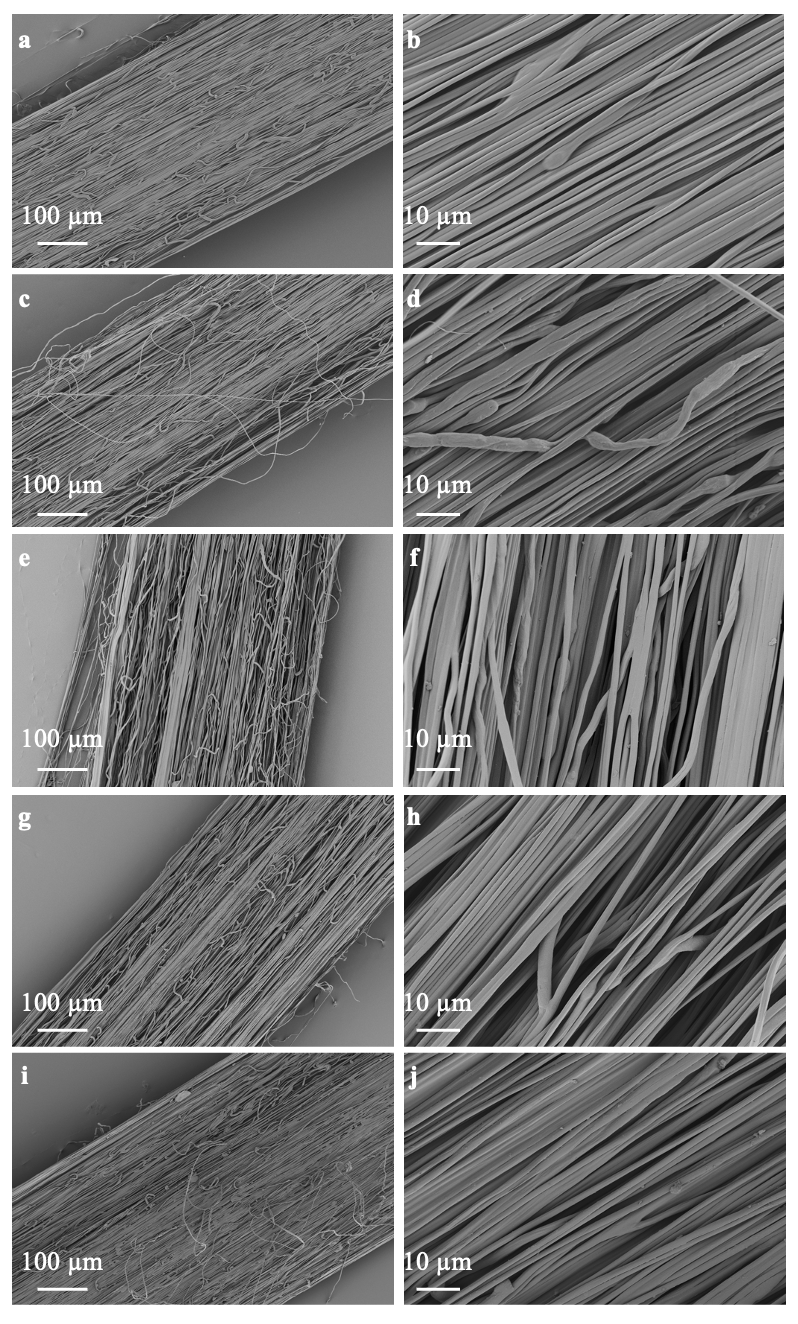


**Fig. S2 SEM characterisation of stretched filaments during 12-month *in vitro* degradation.** SEM images of stretched filament at different time points: month 0 (**a, b**), month 1 (**c, d**), month 3 (**e, f**), month 6 (**g, h**), month 12 (**i, j**). Scale bar: 100 µm in **a, c, e, g, i.** Scale bar: 10 µm in **b, d, f, h, j.**


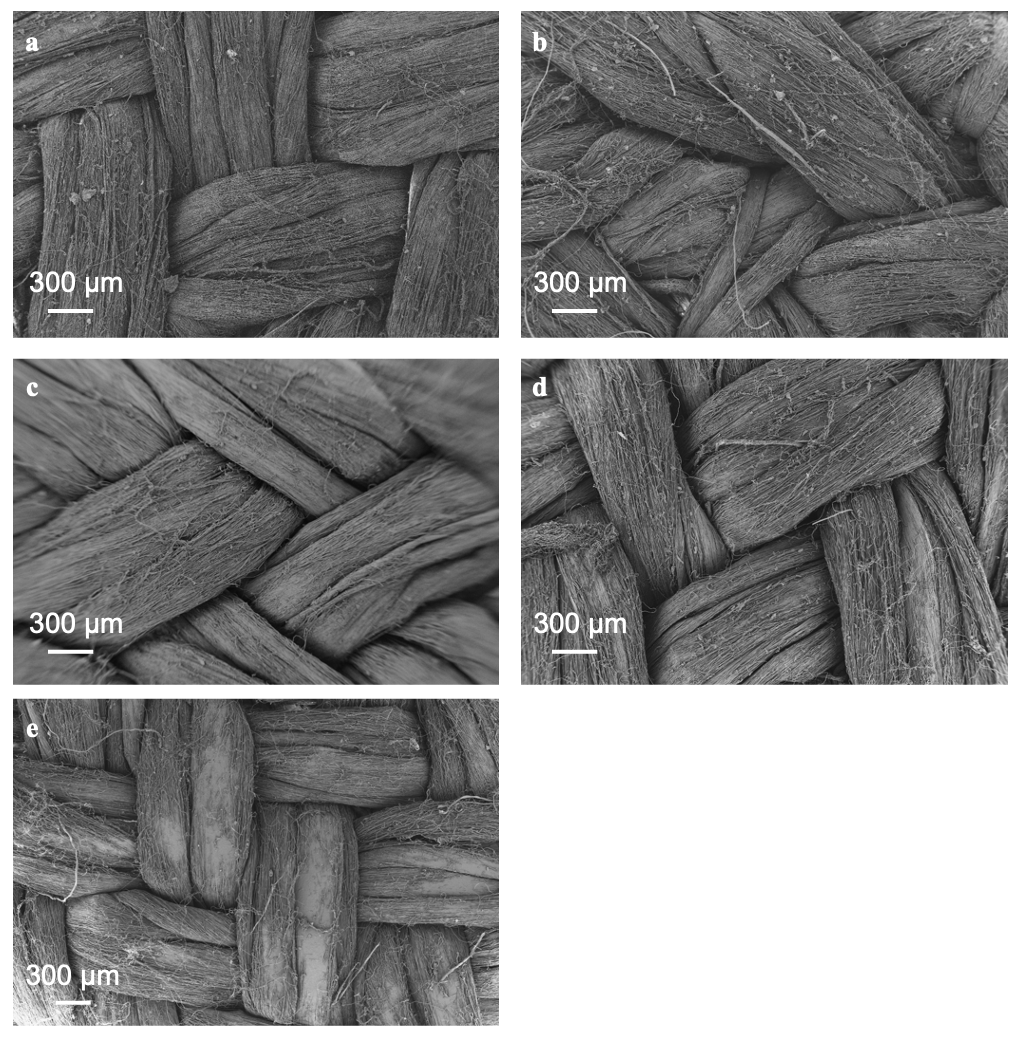


**Fig. S3** **SEM characterisation of braided ES-Lig during 12-month *in vitro* degradation.** SEM images of ES-Lig surface at different time points: month 0 (**a**), month 1 (**b**), month 3 (**c**), month 6 (**d**), month 12 (**e**). Scale bar: 300 µm.


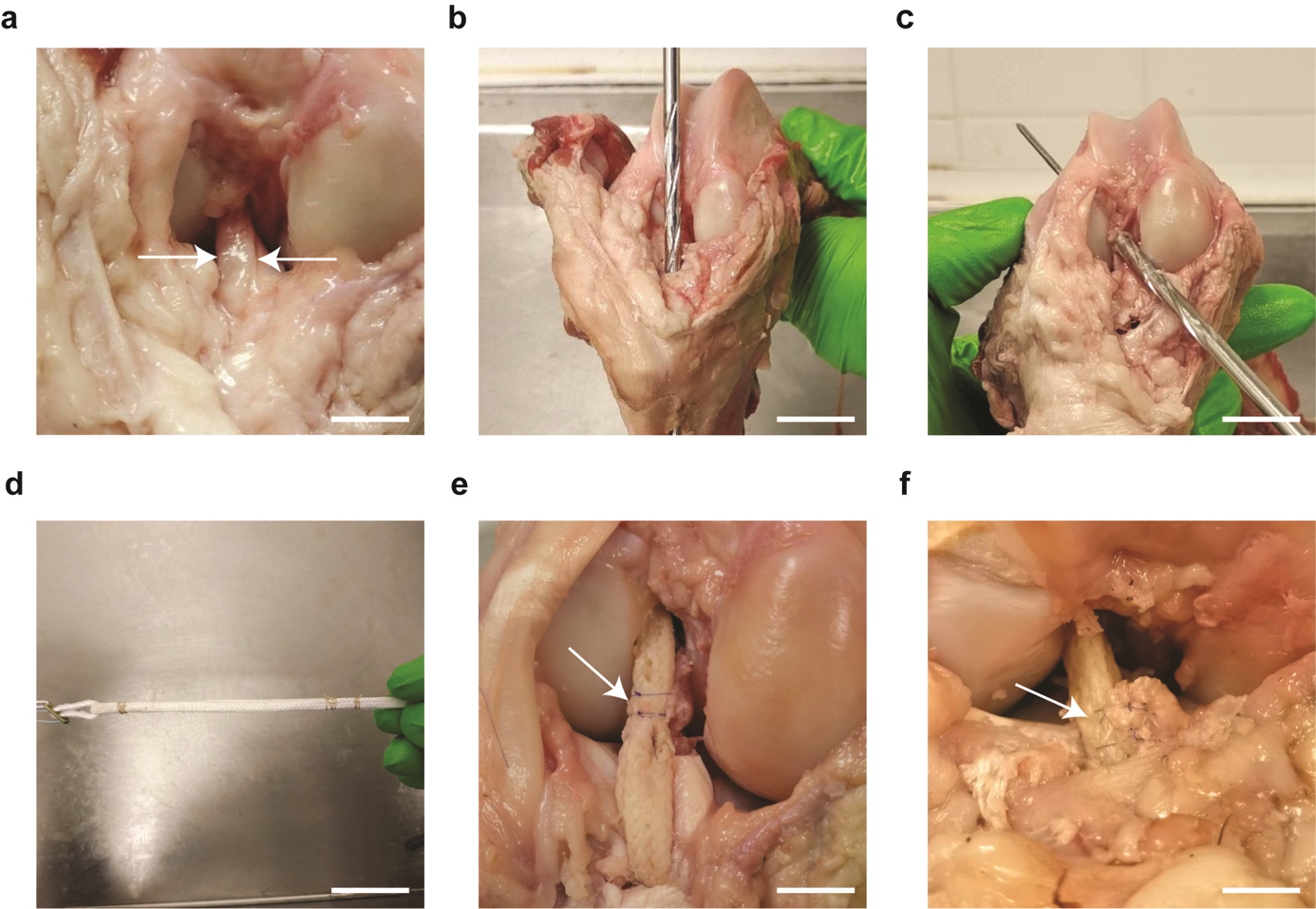


Fig. S4 **Surgical technique and fixation of ES-Lig in sheep cadaveric limbs**. **a,** Exposure and transection of the native ACL (white arrow). Scale bar, 10 mm. **b,** Creation of the femoral tunnel. Scale bar, 30 mm. **c,** Creation of the tibial tunnel. Scale bar, 30 mm. **d,** Integration of ES-Lig with an adjustable-loop cortical suspension device (Endobutton, Smith & Nephew) for femoral fixation. Scale bar, 100 mm. **e,** Securement of the ACL remnant tissue (white arrow) with ES-Lig on the femoral side. Scale bar, 10 mm. **f,** Securement of the ACL remnant tissue (white arrow) with ES-Lig on the tibial side. Scale bar, 10 mm.


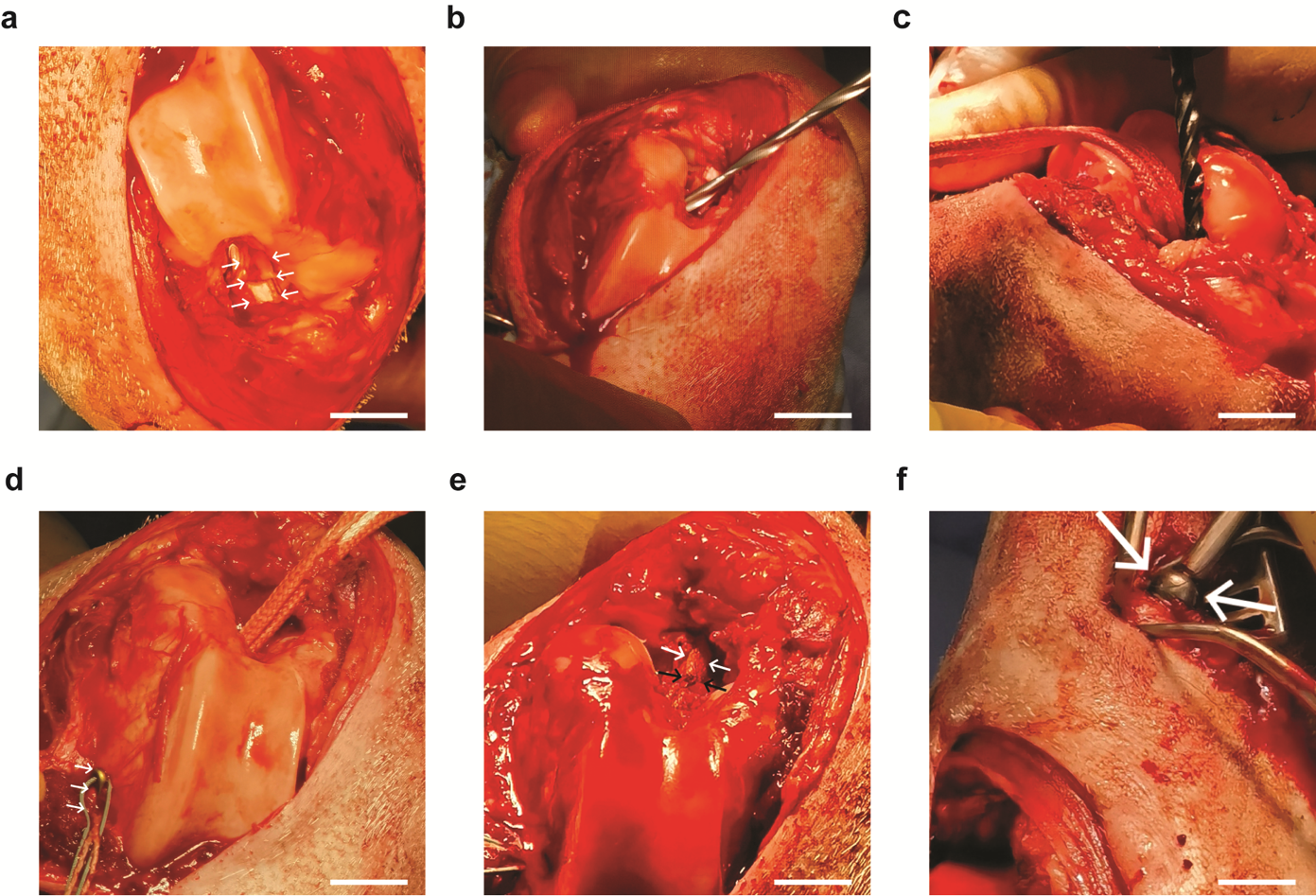


Fig. S5 **Surgical technique and fixation of ES-Lig in an *in vivo* sheep model**. **a,** Midsubstance cutting of the native ACL (white arrow). **b,** Creation of the femoral tunnel. **c,** Creation of the tibial tunnel. **d,** Fixation of the femoral side using the Endobutton (white arrow). **e,** Fixation of the tibial side using the interface screw (white arrow). **f,** Securement of the ACL remnant tissue (black arrow) with the ES-Lig (white arrow), ensuring integration with native tissue. Scale bar, 20 mm.


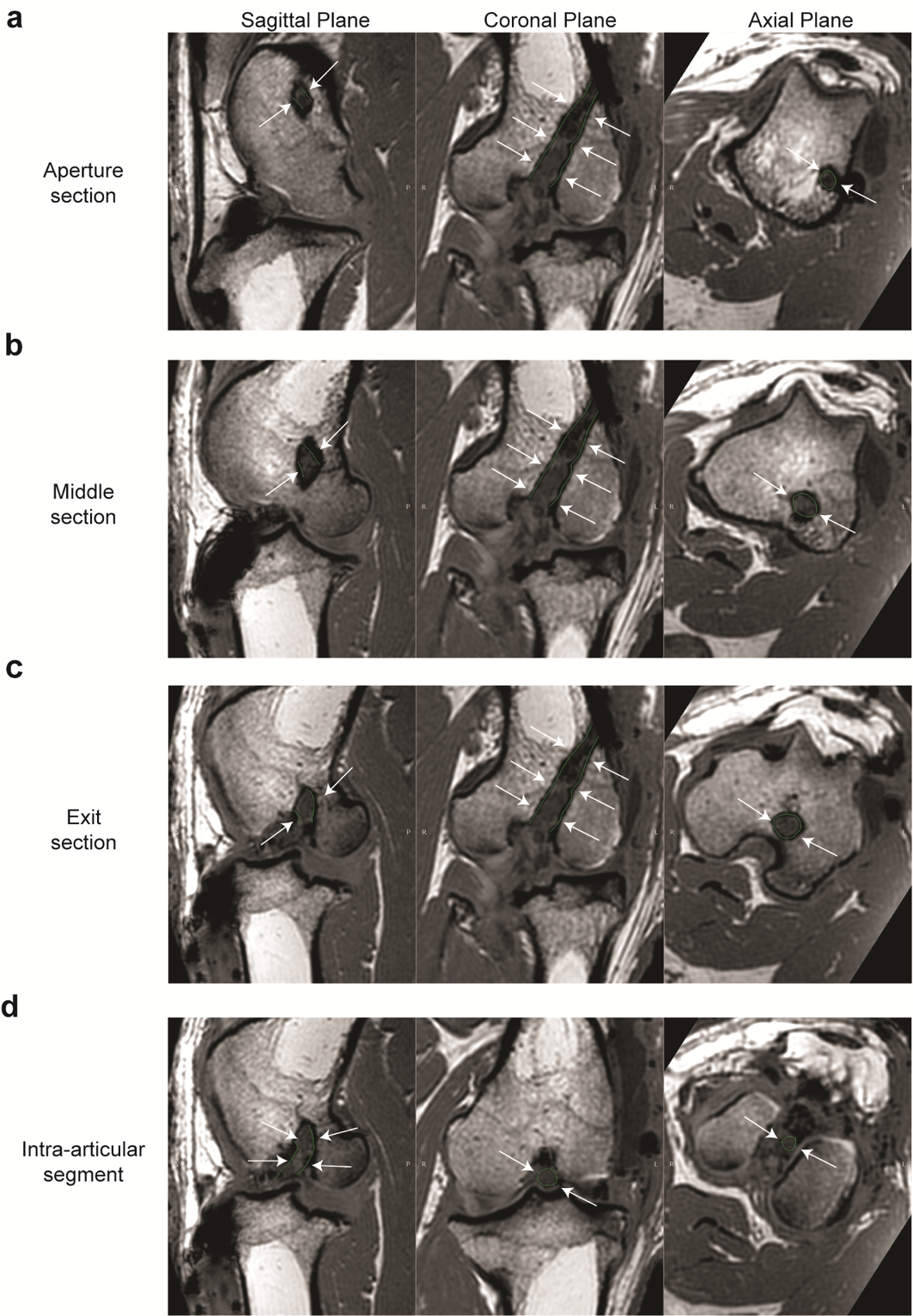


Fig. S6 **ES-Lig MRI after 10-week implantation in an ovine model. a,** ES-Lig within the femoral bone tunnel near the tunnel exit. **b,** ES-Lig within the mid-portion of the femoral bone tunnel. **c,** ES-Lig within the femoral bone tunnel near the joint cavity exit. **d,** Intra-articular portion of ES-Lig. **Note:** The tibial side is not visible due to imaging artifacts caused by the interface screw.


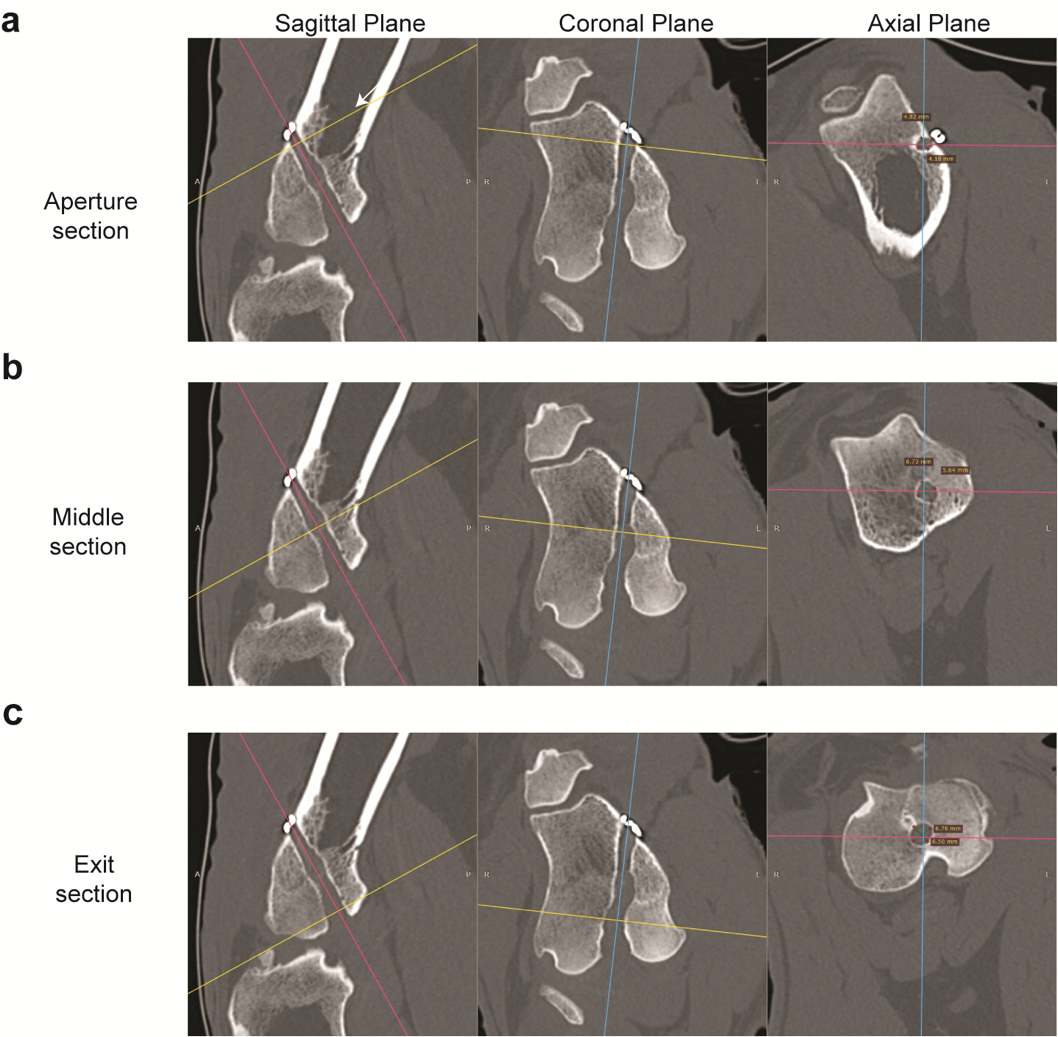


Fig. S7 **Measurement of the femoral bone tunnel diameter at the aperture, middle, and exit in CT**. **a,** The femoral bone tunnel near the aperture (tunnel entry). **b,** The mid-portion of the femoral bone tunnel. **c,** The femoral bone tunnel near the exit (joint cavity side). Measurement of the femoral bone tunnel diameter at the aperture, middle, and exit is shown. The bone tunnel circular plane is oriented perpendicular to the long axis of the femoral tunnel. Tunnel diameters were calculated as the average of two orthogonal diameters (D_1_ and D_2_), representing the long and short axes of the elliptical cross-section. Tunnel diameter = (D_1_ + D_2_) / 2.


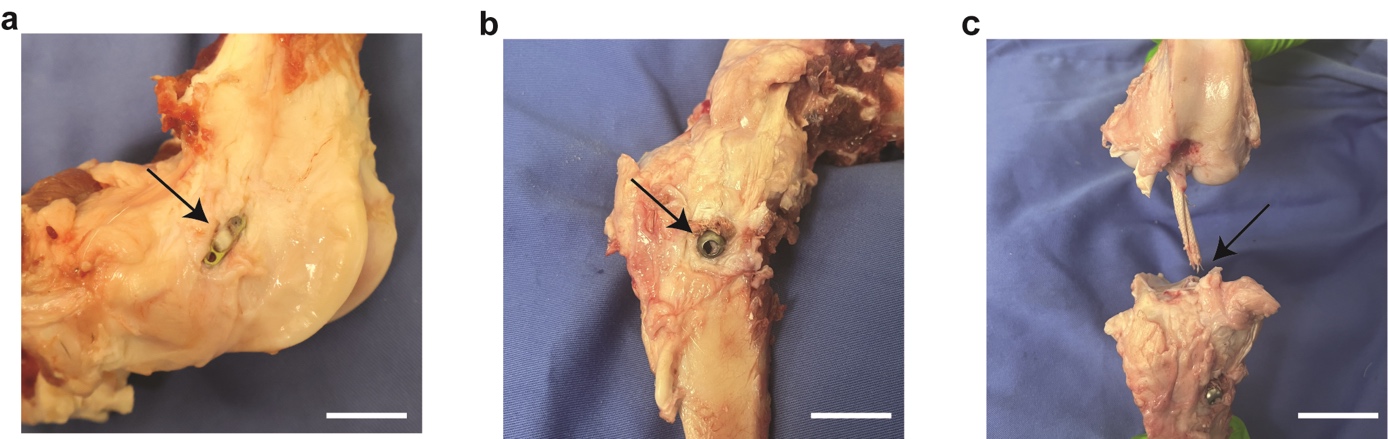


Fig. S8 **ES-Lig fixation and failure mechanism after 10-week implantation in an ovine model**. **a,** Endobutton fixation of ES-Lig at the femoral side. Scale bar, 20 mm. **b,** Interface screw fixation of ES-Lig at the tibial side. Scale bar, 20 mm. **c,** Failure mechanism during the pull-out test. The ES-Lig graft failed due to tibial-side pullout. Scale bar, 10 mm.
